# Gait Analysis Using an Artificial Intelligence-Based Motion Capture System With a Single Smartphone Camera

**DOI:** 10.7759/cureus.87837

**Published:** 2025-07-13

**Authors:** Takuya Usami, Masaya Kisohara, Kazuki Nishida, Daishiro Koboyashi, Ruido Ida, Kohki Matsubara, Haruhiko Tokuda, Nobuyuki Suzuki, Hideki Murakami, Gen Kuroyanagi

**Affiliations:** 1 Department of Orthopedic Surgery, Nagoya City University Graduate School of Medical Sciences, Nagoya, JPN; 2 Department of Radiology, Nagoya City University Graduate School of Medical Sciences, Nagoya, JPN; 3 Center for Advanced Medicine and Clinical Research, Nagoya University Hospital, Nagoya, JPN; 4 Research and Development, Sportip Inc., Tokyo, JPN; 5 Department of Rehabilitation Medicine, Nagoya City University Graduate School of Medical Sciences, Nagoya, JPN; 6 Department of Rehabilitation Medicine, Nagoya City University Mirai Kousei Hospital, Nagoya, JPN; 7 Department of Rehabilitation Medicine, Inabe General Hospital, Inabe, JPN; 8 Department of Metabolic Research, Research Institute, National Center for Geriatrics and Gerontology, Obu, JPN; 9 Department of Clinical Laboratory, National Center for Geriatrics and Gerontology, Obu, JPN

**Keywords:** artificial intelligence, gait analysis, iphone, motion capture, smartphone camera, sportip motion 3d

## Abstract

Introduction: Motion capture is widely used to analyze human gait and enables measurement of various biomechanical parameters. However, conventional infrared-based motion-capture systems are expensive and require a large amount of space, making them difficult to implement in many facilities. Recently, artificial intelligence (AI) has been applied in numerous medical fields, including gait analysis. This study aimed to evaluate the effectiveness of an AI-based motion capture system using a single smartphone camera compared to a conventional infrared-based motion capture system.

Methods and analysis: Twenty-two straight walks of healthy volunteers were simultaneously captured using a smartphone (iPhone X^®^, Apple Inc., Cupertino, CA) placed on the right side of the participants (Group AI) and an infrared-based motion capture system (Group M). In Group AI, gait videos were evaluated by the Sportip Motion 3D AI-based motion capture system (Sportip Inc., Tokyo, Japan). The same walking cycles were analyzed for both methods. Gait parameters, including gait velocity, gait cycle time, step length, and flexion angles of the hip and knee joints, were compared between the two groups.

Results: The shapes of the hip and knee flexion angle graphs in Group AI were similar to those in Group M. Variables, such as gait velocity, bilateral step length, and maximum flexion angle of the hip and knee joints, showed high accuracy. Most variables showed high correlation coefficients (gait velocity, r = 0.94; right and left step lengths, r = 0.91 and 0.93; right and left maximum flexion angle of the hip joint, r = 0.87 and 0.71; knee joint, r = 0.84 and 0.93; right and left minimum flexion angles of the hip joint, r = 0.73 and 0.75). However, low correlation coefficients were observed in gait cycle time (r = 0.68) and minimum knee flexion angle (right and left, r = 0.30 and 0.47).

Conclusion: Our findings suggest that an AI-based motion capture system using a single smartphone camera may provide reliable gait parameters for certain applications.

## Introduction

Gait parameters, such as walking speed, stride length, and hip and knee joint angles, are important and widely recognized in assessing human gait [1,2]. These parameters were acquired using conventional infrared and marker-based motion capture systems. However, marker-based motion capture systems have limitations, such as high costs, space requirements, and the need for specialized skills to operate. Sportip Motion 3D (Sportip Inc., Tokyo, Japan) is an artificial intelligence (AI)-based motion capture system that uses conventional neural networks to learn from over 700,000 human images and the Microsoft COCO dataset [3], which contains a variety of poses captured in various environments and clothing styles. Sportip Motion 3D has already been widely used in several applications, including the posture estimation smartphone software Sportip Pro and the sports motion analysis software program Sportip Motion. Previous studies have demonstrated the accuracy of Sportip Motion 3D during squatting and have shown that the lower limb joint angles provided by Sportip Motion 3D are similar to those obtained by conventional infrared and marker-based motion capture systems (VICON®, Vicon Motion Systems, Oxford, UK) [4]. However, there is no study showing actual human gait analysis using Sportip Motion 3D.

Smartphones are currently used by 60% of the global population. iPhone® (Apple Inc., Cupertino, CA) is one of the world's best-selling smartphones and has been used in several AI-based gait analysis studies [5-8]. In comparing smartphone-based AI motion capture with conventional systems, it has been reported that a smartphone AI motion capture system using the iPhone X can detect the angles of the hip and knee joints in the sagittal plane during countermovement jumps with high accuracy using only a frontal view [7]. It has also been reported that simple videos recorded on a low-cost tablet device and a freely available pose estimation algorithm (OpenPose) enable capturing gait characteristics specific to patients with stroke and Parkinson's disease [8]. However, no studies have been conducted using a single smartphone camera to capture a straight walk. In this study, we compared gait analysis results obtained with a single smartphone camera and Sportip Motion 3D with those obtained using a conventional infrared-based motion capture system.

Therefore, the objectives of this study were to develop an AI-based model for gait analysis using smartphone video data, evaluate the accuracy of the AI model compared to traditional gait analysis techniques, and assess the feasibility of smartphone-based gait assessment in real-world conditions.

## Materials and methods

Ethics

This study was approved by the ethics committee of Inabe General Hospital (2022-4). Written informed consent was obtained from all participants prior to the study.

Study design and patients

**Figure 1:**
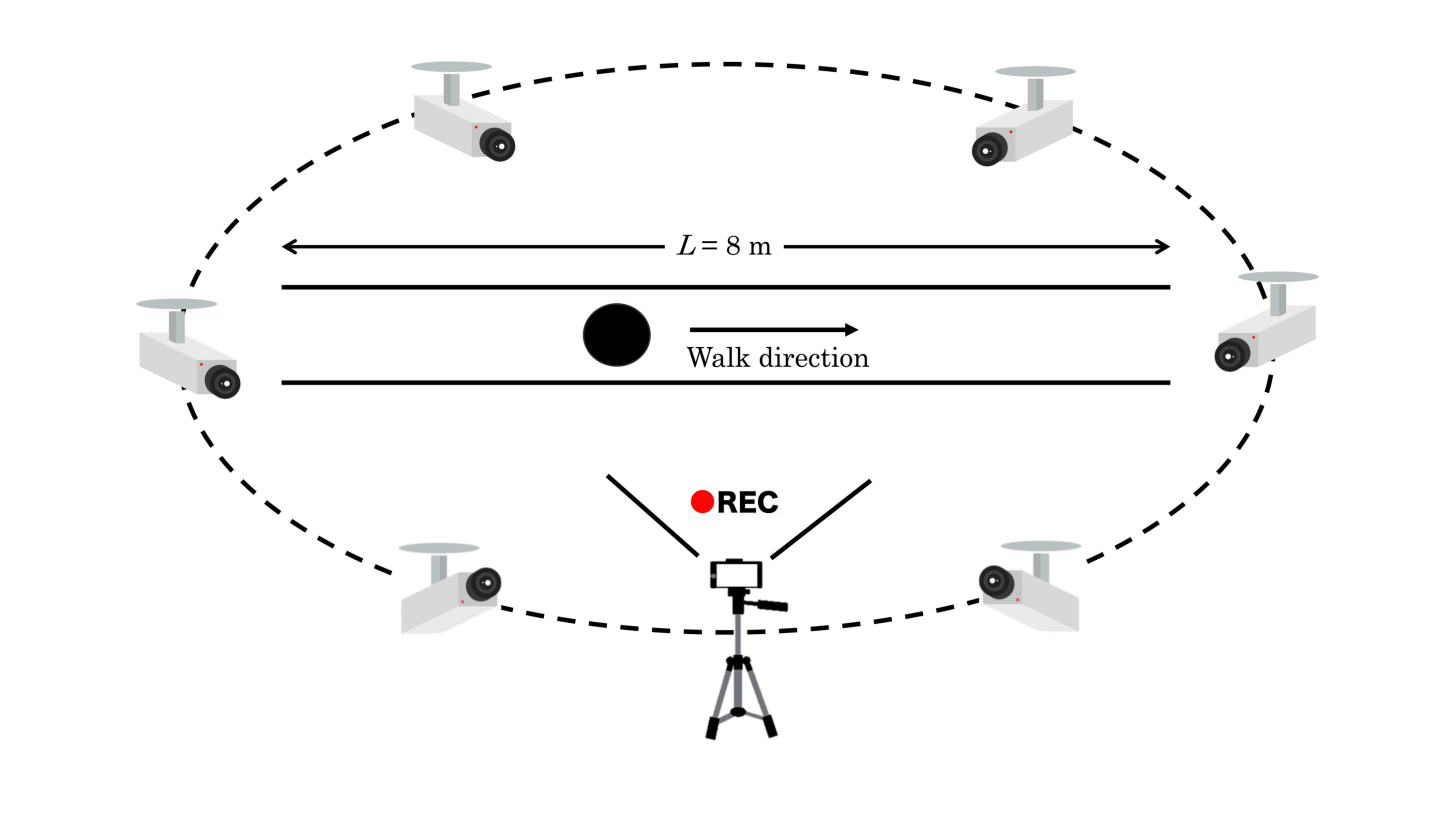
Overview of gait analysis. Walks were captured using an iPhone X from the right side of the participant (Group AI), and six motion captures (MA-3000) were fixed on the ceiling (Group M). The participants walked along a straight line (Figure 1). The walks were also captured by a plantar pressure sensing device (Walk Way MV-1000) (Figure 2).

**Figure 2:**
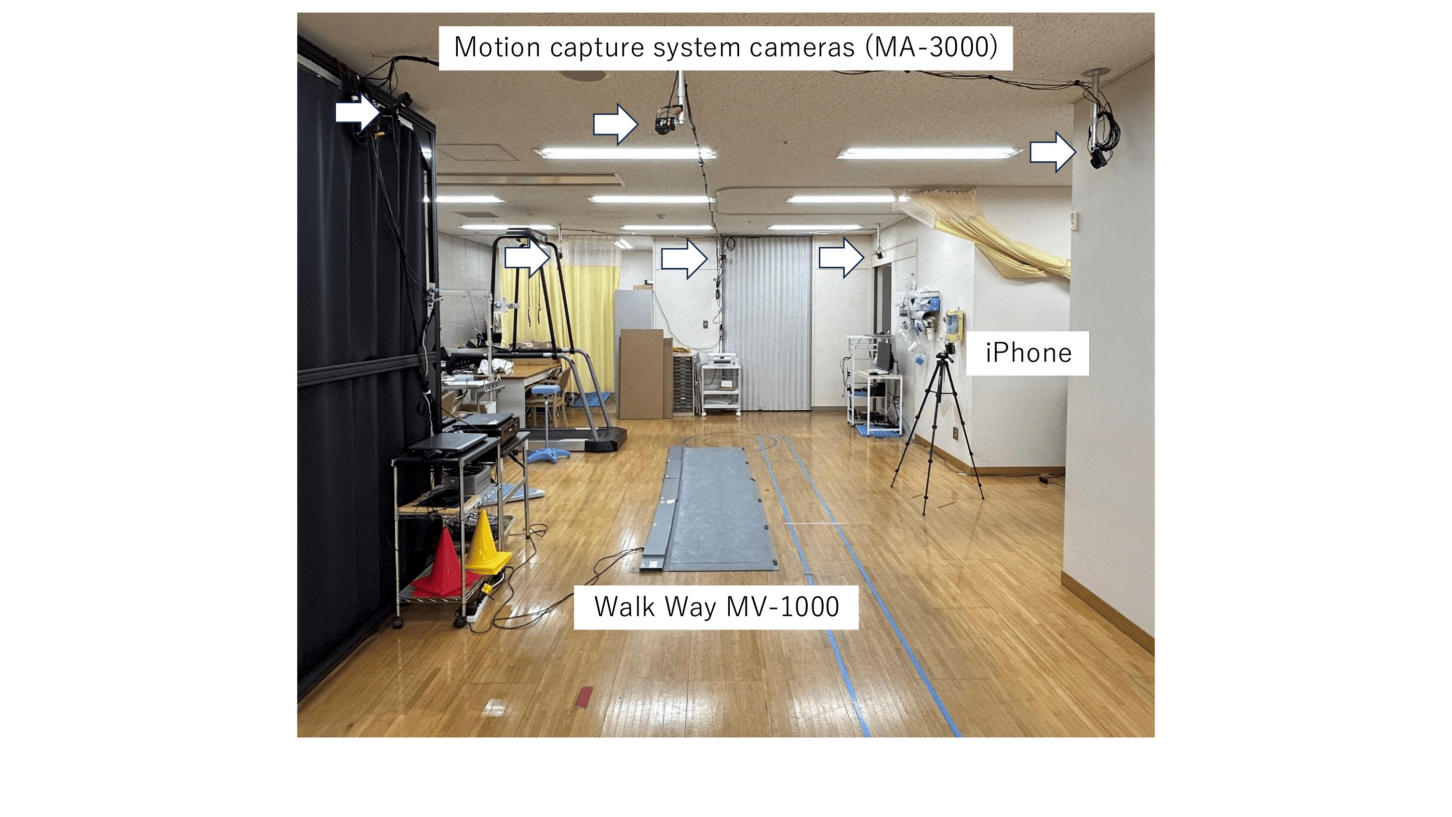
Overview of gait analysis. Walks were captured using an iPhone X from the right side of the participant (Group AI), and six motion captures (MA-3000) were fixed on the ceiling (Group M). The participants walked along a straight line (Figure 1). The walks were also captured by a plantar pressure sensing device (Walk Way MV-1000) (Figure 2).

Healthy male volunteers (n = 3; age: 44.7 ± 12.5 years old, height: 164.7 ± 13.4 cm, weight: 65.7 ± 9.0 kg, BMI: 22.9 ± 3.0 kg/m2) participated in this study. Gait analysis was performed under the same lighting conditions. The participants wore identical clothing. Twenty-four straight walks were simultaneously recorded using two methods: an iPhone X® and an infrared-based motion capture system MA-3000 (ANIMA Inc., Tokyo, Japan). The ground truth was defined using an infrared-based motion capture system (MA-3000), which is a clinically validated system. Additionally, a plantar pressure sensing device, Walk Way MV-1000® (ANIMA Inc.), was also used to validate the spatial parameters and gait cycle time. However, since the current study was not designed for binary classification tasks (e.g., distinguishing normal from abnormal gait), performance metrics such as sensitivity, specificity, F1-score, and receiver operating characteristic (ROC) curves were not applicable. Instead, we focused on evaluating the agreement between Group M and Group AI using correlation coefficients and Bland-Altman plots. In this study, two walking trials could not be properly recorded because some of the infrared markers were not detected. Therefore, we excluded these two walking trials. The iPhone X was placed horizontally on the right side of the participant at a height of 80 cm with a tripod. Regarding addressing potential variations in camera position and lens distortion in a single-camera setup, we used a tripod and carefully checked the camera position, height, and angle during video capture to minimize these errors and ensure repeatability. Then, the gait was captured at a resolution of 3840 × 2160 pixels and 60 frames per second (fps). Each walking cycle was synchronized for analysis, and the same gait cycle was selected for both methods based on the video captured by the iPhone X. Additionally, we chose a gait cycle where the participant was always located near the center of the video frame to minimize the effects of lens distortion. The video captured by iPhone X was edited using Adobe Premiere Pro (Adobe Inc., San Jose, CA). Preprocessing involved trimming the video from heel strike to the subsequent heel strike using Adobe Premiere Pro, maintaining consistent resolution and frame rate. The selected frames were then exported at the original resolution and frame rate as the original recording. This segment was then analyzed using Sportip Motion 3D. The MA-3000 has six infrared high-speed cameras fixed to the ceiling surrounding the walk, capturing at 100 fps (Figures 1, 2). The data obtained by the MA-3000 were exported to CSV files containing adjusted joint angle data and a summary of the gait parameters over one gait cycle.

AI model and training dataset

Sportip Motion 3D employs a top-down pose estimation approach based on the high-resolution network (HRNet) architecture. HRNet is a deep learning model that maintains high-resolution representations throughout the network and is widely used for accurate human pose estimation. HRNet outperformed OpenPose in keypoint detection accuracy on the COCO dataset, demonstrating the advantages of a top-down approach for single-person pose estimation [9]. The AI model was trained using the Microsoft COCO dataset [3] and over 700,000 human motion capture datasets collected from university students. The dataset includes a diverse range of activities such as daily living motions, general sports movements, training exercises, range-of-motion tests, and sports activities, including baseball, basketball, golf, tennis, yoga, and track and field, as well as walking and static postures.

Data analysis

The video was analyzed using Sportip Motion 3D on a Linux PC to estimate the positions of the head, sternum, bilateral hips, knees, ankles, toes, and heels. The angle of the hip joint was calculated using two 3D coordinate vectors of the hip-to-sternum and hip-to-knee. The knee joint angle was determined using knee-to-hip and knee-to-ankle vectors. Spatial parameters were calculated based on the participants’ height. The parameters obtained from the Sportip Motion 3D were labeled as Group AI. Stick skeletal videos were generated using Sportip Motion 3D software by connecting each joint position.

As for gait analysis using a wall-mounted motion capture system, infrared reflective markers were placed on the neck, posterior and anterior superior iliac spines, greater trochanter, femoral lateral condyle, lateral malleolus, and fourth metatarsal. Data captured by the MA-3000 were analyzed using the software MD-1000 (ANIMA Inc.) to obtain gait parameters, including gait velocity, gait cycle time, bilateral step length, maximal and minimum flexion angles of the hip joint, and maximal and minimum flexion angles of the knee joint. The parameters obtained from the MD-1000 were labeled as Group M.

Statistical analysis

Heat maps were used to visualize Pearson’s correlation coefficients between each variable. Scatter plots and Bland-Altman plots between groups AI and M were generated using gait velocity, step length, maximum left and right flexion angles of the hip joint, and maximum flexion angle of the knee joint as variables. All visualizations were conducted using Python (version 3.8.10, Python Software Foundation, Wilmington, Delaware) and the Seaborn Library (version 0.11.1). Continuous variables were presented as mean ± standard deviation. Variables in Group M were subtracted from those in Group AI, and the differences were analyzed using a paired t-test. Pearson’s correlation coefficients were calculated for both groups. The R statistical package (version 3.5.1, R Foundation for Statistical Computing, Vienna, Austria) was used for all statistical analyses. A p-value of < 0.05 was considered statistically significant.

## Results

Visualization and comparison of hip and knee joint angles using AI and a conventional infrared motion capture system

**Figure 3:**
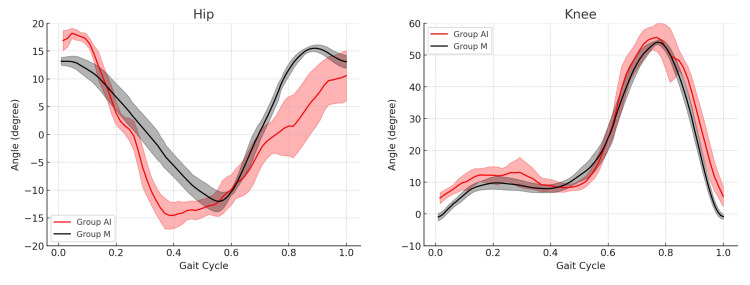
Graph of hip and knee angle gait over one gait cycle (participant 3). The shapes of the gait graph were similar in groups AI and M. The bold line represents the mean angle of all walks. The width of the line is one standard division.

The graphs of the hip and knee joint angles over one gait cycle showed similarities between groups AI and M (Figure 3).

Gait and hip and knee joint parameters and correlation coefficients between AI and a conventional infrared-based motion capture system

The variables in Group M were subtracted from those of Group AI, and the Pearson’s correlation coefficients between groups AI and M are summarized in Tables 1, 2. The results for each of the variables were as follows: gait velocity (0.069 ± 0.32, t = -0.911, p = 0.374; r = 0.94, p < 0.001), gait cycle time (0.0051 ± 0.030, t = -0.727, p = 0.476; r = 0.68, p = 0.002), right step length (5.06 ± 2.53, t = -8.483, p < 0.001; r = 0.91, p < 0.001), left step length (−11.43 ± 30.40, t = -6.346, p < 0.001; r = 0.93, p < 0.001), right maximum flexion angle of the hip joint (−3.91 ± 1.48, t = 11.24, p < 0.001; r = 0.87, p < 0.001), left maximum flexion angle of the hip joint (−3.29 ± 1.99, t = 7.007, p < 0.001; r = 0.71, p < 0.001), right minimum flexion angle of the hip joint (9.86 ± 3.85, t = -10.88, p < 0.001; r = 0.73, p < 0.001), left minimum flexion angle of the hip joint (5.39 ± 13.28, t = -6.969, p < 0.001; r = 0.75, p < 0.001), right maximum flexion angle of the knee joint (−3.31 ± 2.52, t = 5.580, p < 0.001; r = 0.84, p < 0.001), left maximum flexion angle of the knee joint (1.78 ± 2.23, t = -3.388, p = 0.003; r = 0.93, p < 0.001), right minimum flexion angle of the knee joint (−6.13 ± 3.96, t = 6.571, p < 0.001; r = 0.30, p = 0.206), and left minimum flexion angle of the knee joint (−4.58 ± 1.53, t = 12.72, p < 0.001; r = 0.47, p = 0.040), respectively (Table 1).

**Table 1:** Difference of variables obtained using infrared-based motion capture (Group M) and Sportip Motion 3D (Group AI) systems. FA, flexion angle; Max, maximum; Min, minimum. † Based on a paired t-test. * p < 0.05, ** p < 0.01, *** p < 0.001.

Variables	Difference between AI and motion capture	p-value^†^	t-score
Gait velocity (mean ± SD, km/h)	0.069 ± 0.32	0.374	-0.911
Gait cycle time (mean ± SD, seconds)	0.0051 ± 0.030	0.476	-0.727
Right step length (mean ± SD, cm)	5.06 ± 2.53	<0.001^***^	-8.483
Left step length (mean ± SD, cm)	−11.43 ± 30.40	<0.001^***^	-6.346
Right Max FA of the hip joint (mean ± SD, degrees)	−3.91 ± 1.48	<0.001^***^	11.24
Left Max FA of the hip joint (mean ± SD, degrees)	−3.29 ± 1.99	<0.001^***^	7.007
Right Min FA of the hip joint (mean ± SD, degrees)	9.86 ± 3.85	<0.001^***^	-10.88
Left Min FA of the hip joint (mean ± SD, degrees)	5.39 ± 3.28	<0.001^***^	-6.969
Right Max FA of the knee joint (mean ± SD, degrees)	−3.31 ± 2.52	<0.001^***^	5.580
Left Max FA of the knee joint (mean ± SD, degrees)	1.78 ± 2.23	0.003^**^	-3.388
Right Min FA of the knee joint (mean ± SD, degrees)	−6.13 ± 3.96	<0.001^***^	6.571
Left Min FA of the knee joint (mean ± SD, degrees)	−4.58 ± 1.53	<0.001^***^	12.72

**Table 2:** Pearson’s correlation coefficients between the infrared-based motion capture (Group M) and Sportip Motion 3D (Group AI) systems. FA, flexion angle; Max, maximum; Min, minimum. * p < 0.05, ** p < 0.01, *** p < 0.001.

Variables	Correlation	Lower limit	Upper limit	p-value
Gait velocity	0.94	0.84	0.98	<0.001^***^
Gait cycle time	0.68	0.32	0.86	0.002^**^
Right step length	0.91	0.78	0.97	<0.001^***^
Left step length	0.93	0.80	0.98	<0.001^***^
Right Max FA of the hip joint	0.87	0.68	0.95	<0.001^***^
Left Max FA of the hip joint	0.71	0.37	0.88	<0.001^***^
Right Min FA of the hip joint	0.73	0.41	1.00	<0.001^***^
Left Min FA of the hip joint	0.75	0.44	0.90	<0.001^***^
Right Max FA of the knee joint	0.84	0.63	0.94	<0.001^***^
Left Max FA of the knee joint	0.93	0.83	0.97	<0.001^***^
Right Min FA of the knee joint	0.30	−0.67	0.18	0.206
Left Min FA of the knee joint	0.47	0.02	0.76	0.040^*^

Heatmap of Pearson's correlation coefficients and scatter plots between AI and a conventional infrared-based motion capture system

**Figure 4:**
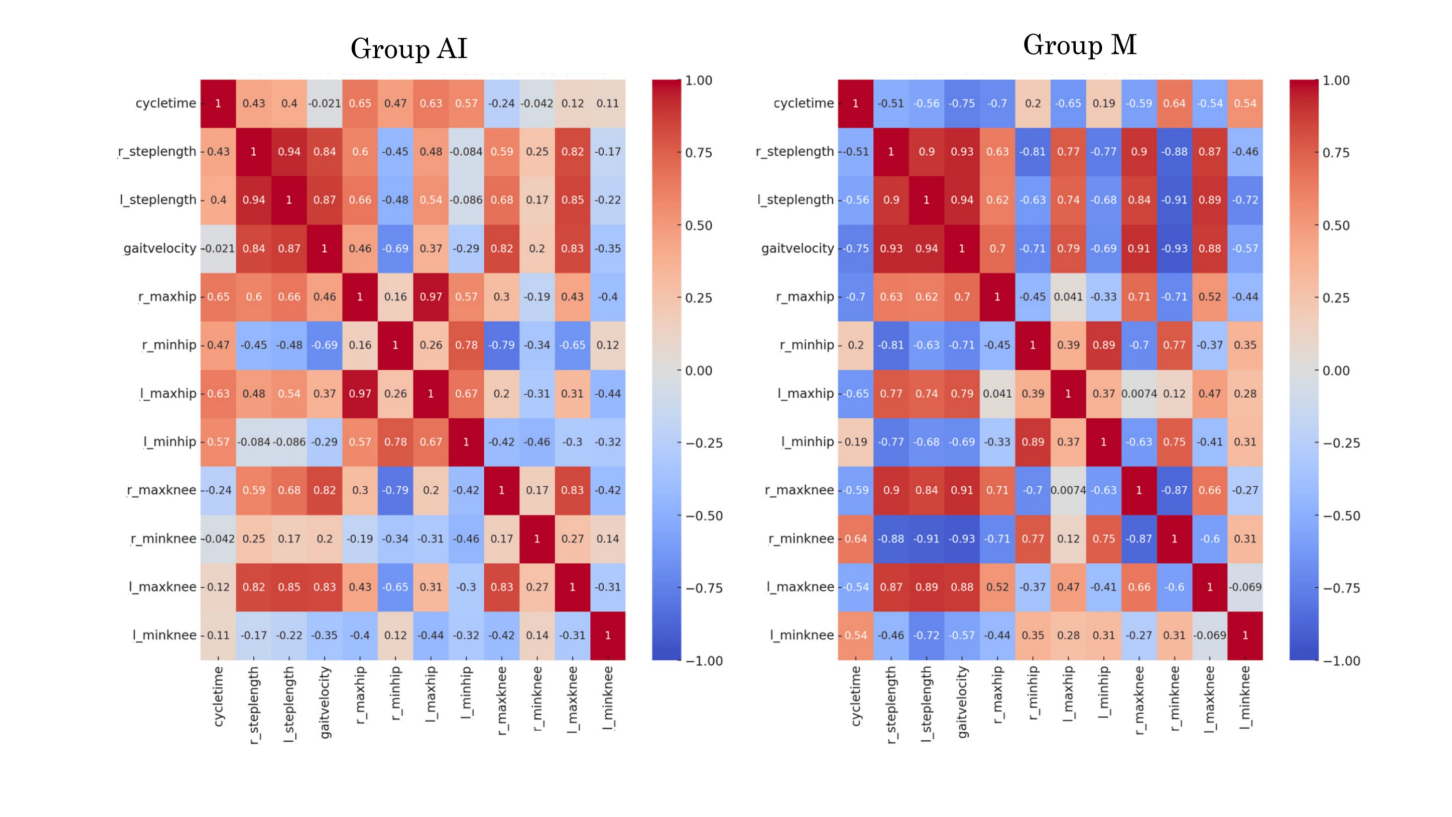
Heat maps displaying Pearson’s correlation coefficients of each pair of variables.

Heatmaps of the Pearson’s correlation coefficients showed similar tendencies between the two groups. However, some variables related to the maximum flexion angle of the hip joint between the right and left sides (0.97° vs. 0.04°) and maximum flexion angle of the knee joint (0.83° vs. 0.66°) showed higher correlation coefficients in Group AI than those in Group M (Figure 4).

**Figure 5:**
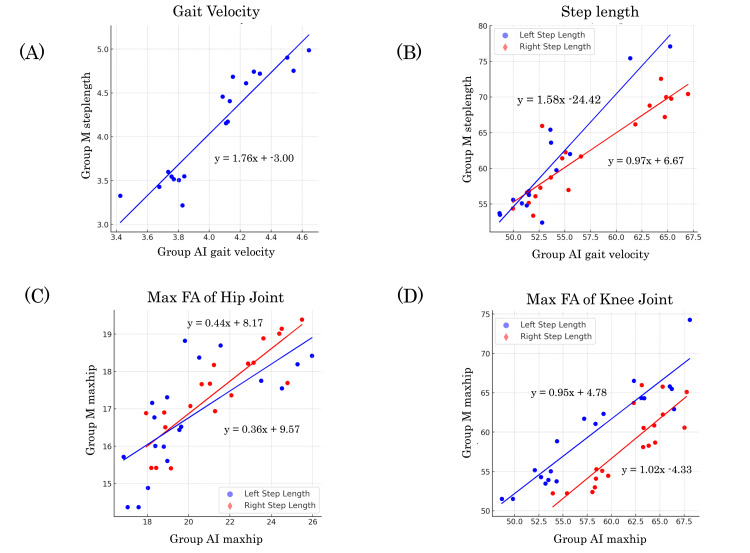
Scatter plots of gait parameters. Scatter plots of (A) gait velocity, (B) step length, (C) maximum flexion angle (FA) of hip angle, and (D) knee angle. The X axis represents the variables in Group AI, and the Y axis represents the variables in Group M. The regression line is drawn as a solid line.

Scatter plots showed that the gait velocity and step length were correlated between groups AI and M. However, the maximum flexion angle of the hip joint, particularly at large angles, did not show a good correlation with Group AI, and the maximum flexion angle of the knee joint differed between the left and right legs (Figure 5).

The fixed errors and the proportional biases

**Figure 6:**
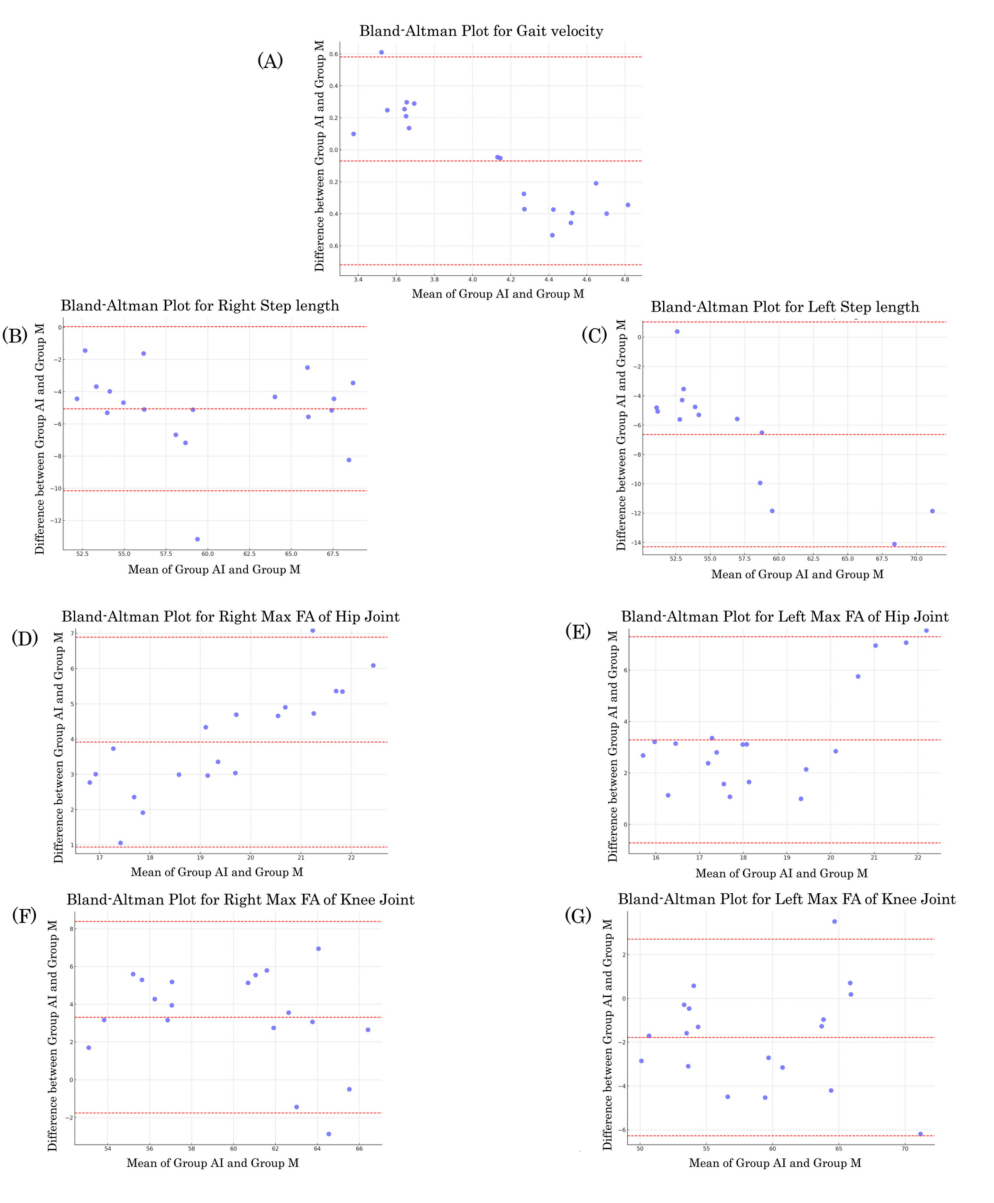
Bland-Altman plot of gait parameters. Bland–Altman plot of (A) gait velocity, (B, C) right and left step lengths, (D, E) right and left maximum flexion angles (FA) of the hip joint, and (F, G) right and left maximum flexion angles of the knee joint. The width of the angle parameters was within 10° between ±1.96 standard deviations (upper and lower red dotted lines).

Bland-Altman plots showed that gait velocity and maximum flexion angle of the hip and knee joints exhibited small fixed errors within 0.1 s for gait velocity and 4° for angle parameters. However, the step-length errors are approximately 5.0-6.5 cm. Gait velocity and right and left maximum flexion angles of the hip joint were proportional. The width of angle parameters was within 10° between ±1.96 standard deviations (Figure 6).

## Discussion

In this study, we compared gait analysis results obtained using a single smartphone camera and AI processing with Sportip Motion 3D with those obtained using a conventional infrared-based motion capture system. To the best of our knowledge, no study has demonstrated the effectiveness of a single smartphone and AI-based motion capture system for gait analysis in healthy individuals. To make this pilot study representative, we used participants with normal gait parameters in gait speed, gait cycle time, step length, and hip and knee joint angles. As a result, this gait analysis method using Sportip Motion 3D (Group AI) showed similar angle graphs in the hip and knee joints compared to the conventional infrared-based motion capture system (Group M), as well as the smooth visualization of the stick skeleton video (Figure 3 and Supplemental Video).

Additionally, we evaluated the gait parameters in Group AI and found high accuracy in gait velocity, bilateral step length, and maximum flexion angle of the hip and knee joints compared with those in Group M (Table 2). Pearson’s correlation coefficients of the parameters between the two groups showed that all variables except for gait cycle time and the minimum flexion angle of the knee joint had statistically significant values >0.7. In this study, the correlation coefficient between gait cycle time (r = 0.68) and minimum knee flexion angle (right: r = 0.30; left: r = 0.47) was low, although other variables showed high correlation coefficients. The gait cycle time may have been affected by the frame rate difference between the systems (Group AI: 60 fps; Group M: 100 fps), which may result in a subtle timing mismatch. For the minimum knee flexion angle, the negative value observed in the Group M suggests that the AI-based motion capture system may have provided a more realistic estimate than the infrared-based motion capture system. Some AI-based motion capture systems use multiple cameras to improve capturing quality and estimation [10,11]. Notably, our single smartphone and AI-based motion capture system could estimate joint trajectories, even when the joint was behind the other leg. Additionally, the variables of the minimum flexion angle of the knee joint seemed to be more practical in Group AI than those in Group M, with negative values, which did not represent a normal human gait [12]. Analysis of the spatiotemporal and angular parameters using scatter and Bland-Altman plots showed that variables, such as gait speed and maximum flexion angle of the hip and knee joints, had small fixed errors. Additionally, the scatter plot analysis showed that gait velocity, step length, and maximum flexion angle of the knee joint in Group AI were highly correlated with those in Group M. Based on these findings, it seems that gait analysis using a single smartphone camera and Sportip Motion 3D provides highly accurate gait analysis compared to the traditional infrared-based motion capture system.

Several studies have shown that marker-less motion capture systems assess gait speed and hip and knee flexion angles [10,11,13-18]. As for the maximal hip and knee flexion angles, the correlation coefficients between gait speed and maximum hip and knee flexion angles obtained in this study were similar to those previously reported for AI-based single digital video camera markerless motion capture systems, with values >0.8. However, these studies were concerned with variations due to lens distortion and experimental setup [13,14]. To make the experiment more consistent, we used the same smartphone and recording environment. Some studies reported the use of a marker-less infrared-based system (Kinect V2®) and showed that the correlation coefficients of hip and knee joint angles were 0.43-0.91 and 0.23-0.94, respectively [15,16]. Additionally, the AI-based marker-less multiple-camera motion capture system Theia3D has been reported to achieve a small average root mean square error (RMSE) of 3.6 cm and 0.96-11.0 degrees for the hip joint, with corresponding values of <2.5 cm and 0.96-6.7 degrees for knee joints [10,11]. In this study, the correlation coefficients for hip angle were moderate, ranging from 0.71 to 0.87. This may be due to the difficulties faced by AI systems in calculating the hip position when using either single or multiple cameras. The inertial sensor-based gait analysis system showed an RMSE of 1.72-5.24 degrees for hip joints and 2.77-3.34 degrees for knee joints when compared to an infrared-based motion capture system. However, inertial sensor-based method faces challenges with calibration for certain patients, reduced accuracy in complex movements, and variability in reliability across conditions and operators [17,18]. In this study, the absolute difference between the maximum flexion angles was 1.78-3.91° of the hip and knee joints. In summary, by comparing our AI-based motion capture system with other marker-less motion capture systems, our system has satisfactory accuracy and correlation coefficients for the spatiotemporal parameters and flexion angles of the hip and knee joints.

AI-based motion capture systems offer several advantages. Infrared-based motion capture systems typically require spatiotemporal calibration to synchronize data captured by multiple cameras [19,20], whereas an AI-based motion capture system does not require expensive equipment, specialized skills, dedicated space, and calibration. The only information requested is the participant’s height to obtain spatial information. Thus, capture could be facilitated regardless of the participant's position. Additionally, we compared variables between the two groups and found that many spatial and angular parameters had smaller standard deviations in Group AI than in Group M (Supplementary Table). Therefore, an AI-based motion capture system not only simplifies the capture process by reducing the need for expensive equipment, complex calibration, and space limitations but also provides more consistent spatial and angular measurements. However, this study was performed under controlled conditions with a fixed camera angle and a small sample of healthy male participants. In real-world settings, key point detection may be affected by occlusions, lighting, and subject factors such as age or assistive device use. Further validation under varied conditions is needed for broader applicability. AI-based motion capture systems offer potential advantages for easy gait monitoring, fall risk assessment in the elderly, and remote rehabilitation. Future studies are also needed to confirm the robustness of the AI system from various angles and possibly with different types of movement, such as turning gait and stair walking. Sportip Motion 3D AI-based gait analysis system is trained on a variety of datasets, including the Activities of Daily Living and COCO datasets, and can be further re-trained on data for elderly people, pathological gait patterns, and complex movements. These future studies will be helpful in verifying the robustness of AI-based gait analysis systems in real clinical practice.

Limitations

Despite the high accuracy and correlation between AI-based motion capture and conventional infrared-based motion capture systems, there are some limitations. First, the system was evaluated using videos captured only from the right side of the participants. Because this system was designed to evaluate participants from all directions, evaluations from other directions are required. Second, we analyzed only three healthy young participants using a single iPhone X, which may introduce bias and affect the reproducibility. To evaluate the accuracy of AI-based motion capture systems, future studies should increase the sample size and consider differences in other smartphone devices.

## Conclusions

This study is a "pilot study" and proposes the potential feasibility of using a single smartphone-based AI motion capture system to estimate gait parameters in healthy individuals. Furthermore, we introduced the potential advantages of the AI-based motion capture system for real-world medical or rehabilitation applications. The gait analysis method using Sportip Motion 3D showed similar angle graphs for the hip and knee joints compared to the conventional infrared-based motion capture system. Additionally, gait velocity, bilateral step length, and the maximum flexion angles of the hip and knee joints showed high accuracy, with strong correlations between the two systems.

Compared to conventional infrared-based motion capture systems, an AI-based motion capture system does not require expensive equipment, specialized skills, dedicated space, or calibration. Therefore, an AI-based motion capture system not only simplifies the capture process by reducing the need for expensive equipment, complex calibration, and space limitations but also provides more consistent spatial and angular measurements.

## Appendices

**Table 3:** Variables obtained using Sportip Motion 3D (Group AI) and infrared-based motion capture (Group M) systems. FA, flexion angle; Max, maximum; Min, minimum.

Variables	AI	Motion capture
Gait velocity (mean ± SD, km/h)	4.05 ± 0.31	4.14 ± 0.61
Gait cycle time (mean ± SD, seconds)	1.09 ± 0.03	1.10 ± 0.04
Right step length (mean ± SD, cm)	56.79 ± 5.66	62.5 ± 6.16
Left step length (mean ± SD, cm)	55.61± 5.20	60.32 ± 7.65
Right Max FA of hip joint (mean ± SD, degrees)	21.72 ± 2.44	17.57 ± 1.28
Left Max FA of hip joint (mean ± SD, degrees)	20.67 ± 3.06	16.83 ± 1.39
Right Min FA of hip joint (mean ± SD, degrees)	−23.16 ± 5.28	−13.35 ± 3.34
Left Min FA of hip joint (mean ± SD, degrees)	−19.95 ± 4.69	−14.75 ± 3.23
Right Max FA of knee joint (mean ± SD, degrees)	61.92 ± 3.72	58.49 ± 4.69
Left Max FA of knee joint (mean ± SD, degrees)	58.21 ± 5.75	59.87 ± 6.12
Right Min FA of knee joint (mean ± SD, degrees)	2.23 ± 1.54	−3.91 ± 3.23
Left Min FA of knee joint (mean ± SD, degrees)	1.74 ± 1.72	−2.70 ± 1.24
